# P47 Infographics about antibiotics: making facts accessible

**DOI:** 10.1093/jacamr/dlac004.046

**Published:** 2022-02-16

**Authors:** Oliver van Hecke, Joseph Lee, Chris Butler, Michael Moore, Sarah Tonkin-Crine

**Affiliations:** Nuffield Department of Primary Care Health Sciences, University of Oxford, Radcliffe Observatory Quarter, Woodstock Road, Oxford, OX2 6GG, UK; Nuffield Department of Primary Care Health Sciences, University of Oxford, Radcliffe Observatory Quarter, Woodstock Road, Oxford, OX2 6GG, UK; Nuffield Department of Primary Care Health Sciences, University of Oxford, Radcliffe Observatory Quarter, Woodstock Road, Oxford, OX2 6GG, UK; Primary Care and Population Sciences, University of Southampton, Southampton, UK; Nuffield Department of Primary Care Health Sciences, University of Oxford, Radcliffe Observatory Quarter, Woodstock Road, Oxford, OX2 6GG, UK

## Abstract

**Background:**

Public misconceptions about antibiotic use persist despite the efforts of antibiotic awareness campaigns. These campaigns have often followed a top-down approach and have not sought input from the public. Communities need to see antibiotic campaign messages as relevant, accessible and important in order to have an influence on health-seeking behaviour and antibiotic use.

**Objectives:**

To develop a series of evidenced-based infographics (EBIs) on antibiotic use for common infections in children and to evaluate their effectiveness at increasing parents' understanding of antibiotic use and antibiotic resistance.

**Methods:**

There were three phases to this research. In Phase 1 we set out to identify and summarize scientific evidence for the use of antibiotics for three common infections in children (sore throat, acute cough and otitis media). Phase 2 focused on co-design of a series of prototype EBIs for each infection in focus groups with parents of young children and graphic designers, to test the face and content validity. Phase 3 tested the feasibility of EBIs in increasing parents' understanding about antibiotic use and the perceived relevance of antibiotic resistance in an online survey.

**Results:**

We iteratively co-produced 10 prototype EBIs. Parents found the evidence displayed in the EBIs novel and relevant to their families. Parents did not favour EBIs that were too medically focused. The way the information was displayed influenced their understanding. Parents preferred one health message per EBI. We included eight EBIs in a national survey of parents (*n = *998). EBIs improved knowledge by more than a third across the board (34%, IQR 20%–46% *P<*0.001). Respondents confirmed that EBIs were novel and potentially useful, corroborating our focus group findings.

**Conclusions:**

Co-designed EBIs have the potential to succinctly change parents' perceptions about antibiotics for acute respiratory tract infections in children. Further research should test EBIs in real-world settings to assess their reach as a potential public-facing intervention.

